# Synthesis of sensitive oligodeoxynucleotides containing acylated cytosine, adenine, and guanine nucleobases

**DOI:** 10.3390/dna5020025

**Published:** 2025-05-09

**Authors:** Komal Chillar, Rohith Awasthy, Marina Tanasova, Shiyue Fang

**Affiliations:** Department of Chemistry, and Health Research Institute, Michigan Technological University, 1400 Townsend Drive, Houghton, MI 49931, USA

**Keywords:** 2acG, 4acC, 6acA, Dmoc, DNA, modification, oligonucleotide, synthesis

## Abstract

The synthesis of oligodeoxynucleotides (ODNs) containing the base-labile *N*^6^-acetyladenosine (6acA), *N*^2^-acetylguanosine (2acG), and *N*4-methyoxycarbonyldeoxycytidine (4mcC), as well as up to four *N*^4^-acetyldeoxycytidine (4acC) modifications is described. The 1,3-dithian-2-yl-methoxycarbonyl (Dmoc) group was used as the linker for solid phase synthesis, and the methyl Dmoc (meDmoc) group was used for the protection of the *exo*-amino groups of nucleobases. Deprotection and cleavage were achieved under non-nucleophilic conditions, under which the highly sensitive 4acC, 6acA, 2acG, and 4mcC were found completely stable. Among the modified nucleotides, 4acC has been found in nature, and proven beneficial to DNA duplex stability. Although 6acA, 2acG and 4mcC have not been found in nature, a synthetic route to ODNs containing them is expected to facilitate projects aimed at studying their biophysical properties as well as potential for antisense, RNAi, CRISPR, and mRNA therapeutic applications.

## 1. Introduction

**Figure 1.**
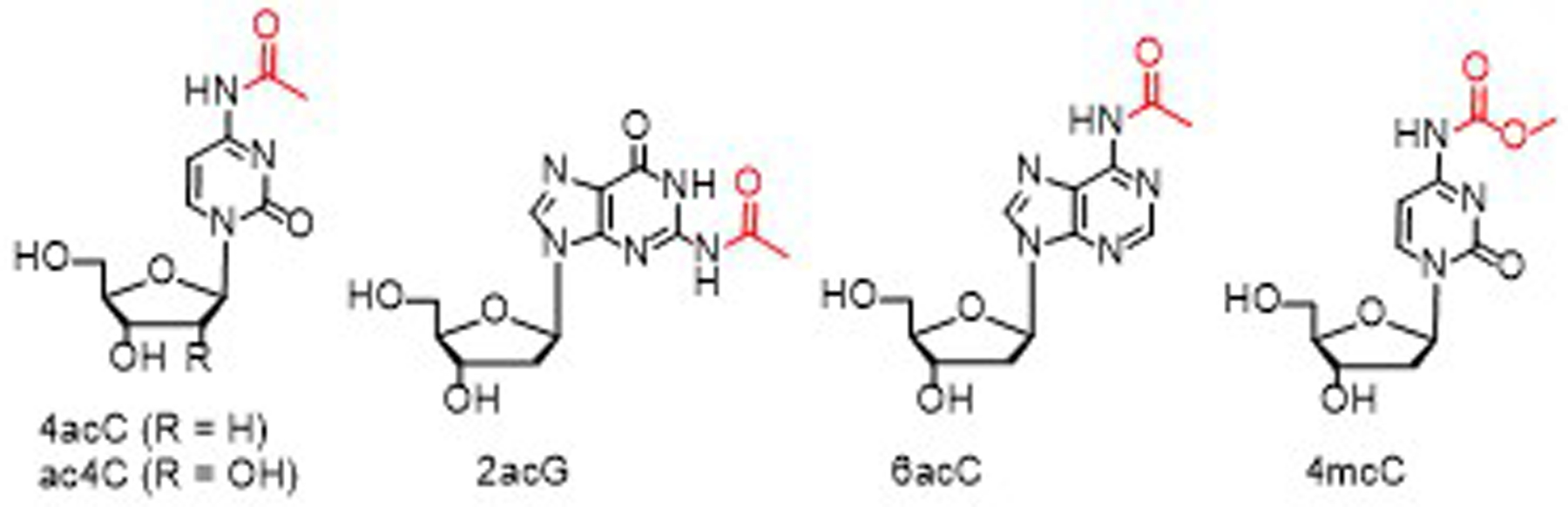
Structure of 4acC, ac4C, 2acG, 6acA, and 4mcC.

Acetylation of the nucleoside cytidine, which gives the *N*^4^-acetylcytidine (ac4C, Figure 1) epitranscriptomic modification, has been observed in RNAs including tRNAs, rRNAs, mRNAs and various regulatory RNAs. It plays a wide range of roles in biological systems. Errors related to the modification have been found to be associated with many human diseases [1–5]. More recently, acetylation of deoxycytidine, which gives the *N*^4^-acetyldeoxycytidine (4acC, Figure 1) epigenetic modification, has also been discovered in the DNA of *Arabidopsis*, rice, maize, mouse, and human. It is mainly located around transcription start sites and positively correlates with gene expression levels [6–8]. Several studies using synthetic oligodeoxynucleotides (ODNs) and oligoribonucleotides (ORNs) have shown that acetylation of cytosine can increase the UV melting temperature of duplex oligonucleotides (ONs) by 1 – 8 °C [9–11]. The knowledge sheds a light on the mechanisms by which ac4C plays roles such as enhancing protein synthesis efficiency in the biological system [12–14]. This shows the significance of the synthesis of ONs containing sensitive modifications such as ac4C and 4acC as well as numerous others [15–18]. However, standard ON synthesis methods requires deprotection and cleavage under strongly basic and nucleophilic conditions, under which ac4C, 4acC and other sensitive groups are unstable. Although several reported methods may be used for the purpose, they have various limitations as discussed earlier [19–22]. For example, some methods can only synthesize ONs that contain thymidine or uridine only and thus do not need nucleobase protection [9, 10]. Some require UV irradiation for cleavage, which can damage ON [11]. Some are limited to the synthesis of short ONs [23, 24]. Therefore, the development of practically useful methods for sensitive ON synthesis without any sequence limitations and with broad sensitive group scope is highly significant.

Our research group recently reported base-labile ODN synthesis using the Dmoc function for linking, and the meDmoc group for the protection of *exo*-amino groups of the nucleobases cytosine, adenine and guanine. Using these protecting and linking strategy, ODN deprotection and cleavage were accomplished under non-nucleophilic and weakly basic conditions [25]. The method enabled us to synthesize ODN sequences containing various sensitive groups including 4acC. In this paper, we report the synthesis of ODNs containing up to four 4acC modifications (ODNs **1a**–**d**, Table 1), as well as those containing the *N*^2^-acetyldeoxyguanosine (2acG, Figure 1, ODN **1e**), *N*^6^-acetyldeoxyadenosine (6acA, Figure 1, **1f**), and *N*^4^-methoxycarbonyldeoxycytidine (4mcC, Figure 1, ODN **1g**) modifications. Unlike ac4C and 4acC, the 6acA, 2acG, and 4mcC as well as ac6A, ac2G and mc4C modifications have not been found in the nature. Our rationale for the synthesis of ODNs containing them includes facilitating projects with aims such as evaluating the potential of the modifications for antisense, RNAi, CRISPR, and mRNA therapeutic applications, wherein the modifications may increase drug cellular stability, improve drug binding affinity, and reduce off-target probability [26–33].

## 2. Materials and Methods

### ODN synthesis, deprotection, cleavage, purification and characterization:

**Figure 2.**
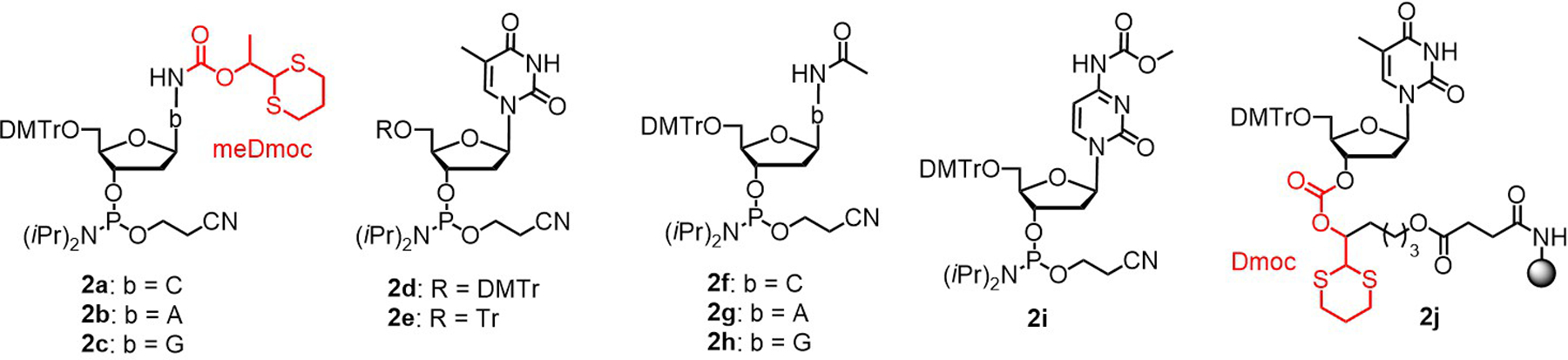
Phosphoramidites and linker for sensitive ODN synthesis using the meDmoc method.

**Scheme 1.**
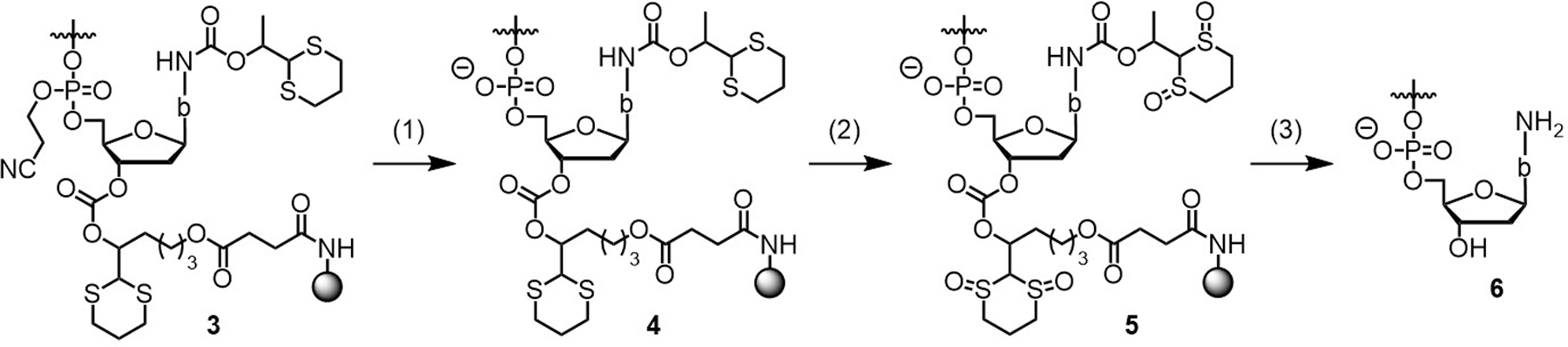
Deprotection and cleavage of ODNs synthesized using meDmoc phosphoramidites and Dmoc linker. The “b” in **3**–**6** represents the nucleobase in **2a**–**j** except that in the cases of **2d**–**e** and **2j**, there is no meDmoc protected *exo*-amino group, in the cases of **2f**–**h**, the *exo*-amine is protected with an acetyl group instead of meDmoc group, and in the case of **2i**, the *exo*-amine is protected with a methoxycarbonyl (mc) group. The “Tr” group at the 5’-end of **3**–**6**, which is introduced with **2e**, is not shown. Conditions for deprotection and cleavage of **3**: (1) 10% DBU in ACN, rt, 15 min. (2) 0.4 M NaIO_4_, pH 4, rt, 4.5 h. (3) 0.05% K_2_CO_3_, pH 8, rt, 5 h.

All ODNs were synthesized on an MerMade 6 synthesizer on the Dmoc support **2j** (Figure 2, 26 µmol/g loading, 20 mg, 0.52 µmol) using phosphoramidite chemistry. Deblocking: DCA (3%, DCM), 90 sec × 2. Coupling: Phosphoramidite (Figure 2, **2a**–**d, 2f**–**i**, and **2e** for incorporating dC, dA, dG, T, 4acC, 6acA, 2acG, 4mcC, and the T at 5′-end of ODN, respectively; 0.1 M, ACN), 4,5-dicyanoimidazole (DCI, 0.25 M, ACN), 90 sec × 3. Capping: 2-Cyanoethyl *N*,*N*,*N′*,*N′*-tetraisopropylphosphoramidite (0.1 M, ACN), DCI (0.25 M, ACN), 60 sec × 3. Oxidation: I_2_ (0.02 M, THF/pyridine/H_2_O, 70:20:10, v/v/v), 40 sec × 3. The 5’-trityl group was kept to assist RP HPLC. The CPG (**3**, Scheme 1) was divided into 5 portions (~0.104 µmol ODN each). One portion was subject to deprotection and cleavage (Scheme 1). Deprotect 2-cyanoethyl groups: The suspension of CPG (**3**, ~0.104 µmol ODN) in the solution of DBU in ACN (1:9, v/v, 1 mL) in a 1.5 mL centrifuge tube was gently shaken at rt for 5 min. The supernatant was removed. The process was repeated two more times. The CPG was washed with ACN (1 mL × 5). This converted **3** to **4**. Oxidize meDmoc and Dmoc: The suspension of **4** in the solution of NaIO_4_ (0.4 M, 1 mL), which has a pH of 4, in a 1.5 mL centrifuge tube was gently shaken at rt for 1.5 h. The supernatant was removed. The process was repeated two times. The CPG was washed with water (1 mL × 5). This converted **4** to **5**. Remove oxidized meDmoc groups: The suspension of **5** in the solution of K_2_CO_3_ (0.05%, 1 mL), which has a pH of 8, in a 1.5 mL centrifuge tube was gently shaken at rt for 5 h. The supernatant was transferred into a 1.5 mL centrifuge tube using a pipette. The CPG was washed with water (150 μL × 5). The combined supernatant and washes were concentrated to ∼50 μL. To the solution was added *n*BuOH (450 μL). After mixing by vortex, ODN was precipitated *via* centrifugation (14.5k rpm, ~14k × g, 15 min). The supernatant was removed leaving deprotected ODN (**6**) in the tube. RP HPLC purification: ODN (**6**) was dissolved in H_2_O (100 μL). A portion (35 μL) was purified with RP HPLC (see supporting information for HPLC conditions). The fractions of the Tr-on ODN were combined and volatiles were removed under vacuum. To the ODN was added AcOH (80%, 1 mL). The mixture was shaken gently at rt for 3 h. Volatiles were evaporated. The residue was dissolved in water (100 μL), and a portion (50 μL) was purified with RP HPLC. The purified Tr-off ODN was analyzed with RP HPLC. The ODNs were quantified using a reported method [34], and analyzed with MALDI MS. The purity of the ODNs was further confirmed using capillary gel electrophoresis and PAGE.

## 3. Results and Discussion

The ODN syntheses were accomplished using phosphoramidites **2a**–**i**, and the linker **2j** (Figure 2). Among them, **2a**–**c** are standard meDmoc phosphoramidites, and **2j** is a standard Dmoc linker. Monomers **2e**, **2g**–**i** are known compounds [10, 35, 36], and they were synthesized in house. The details for the synthesis of **2g**–**i** are provided in the Supporting Information. Monomers **2d** and **2f** were purchased from commercial sources. The syntheses were carried out under conditions similar to those using standard phosphoramidite chemistry except that capping was achieved using 2-cyanoethyl *N*,*N*,*N′*,*N′*-tetraisopropylphosphoramidite and the nucleotide at the 5′-end was incorporated using phosphoramidite **2e**. According to trityl assay, all the phosphoramidites had similar coupling efficiency as standard commercial phosphoramidites. At the end of syntheses, the ODNs can be represented by **3** (Scheme 1), which contained a Tr group (not shown in **3**) introduced by **2e**. Deprotection and cleavage were achieved in three steps (Scheme 1). In the first step, the 2-cyanoethyl groups were removed by washing the CPG with 10% DBU in ACN, which converted **3** to **4**. In the second step, the Dmoc and meDmoc groups were oxidized with NaIO_4_ converting **4** to **5**. In the third step, the oxidized meDmoc and Dmoc groups were cleaved via β-elimination induced by the weak non-nucleophilic base K_2_CO_3_. This gave the fully deprotected ODN with a 5′-Tr group **6** (5′-Tr not shown). All the three steps were convenient to operate because during the first two steps, the ODNs were still on the solid support. Excess reagents and side products could be removed simply by washing. For the third step, the solid support and the ODNs in the supernatant were easy to separate, and the quantity of K_2_CO_3_ was minute, which did not require to be removed before HPLC purification of the ODN. In addition, all the deprotection and cleavage reactions were carried out at room temperature, which ensured stability of sensitive groups on the ODNs.

**Figure 3.**
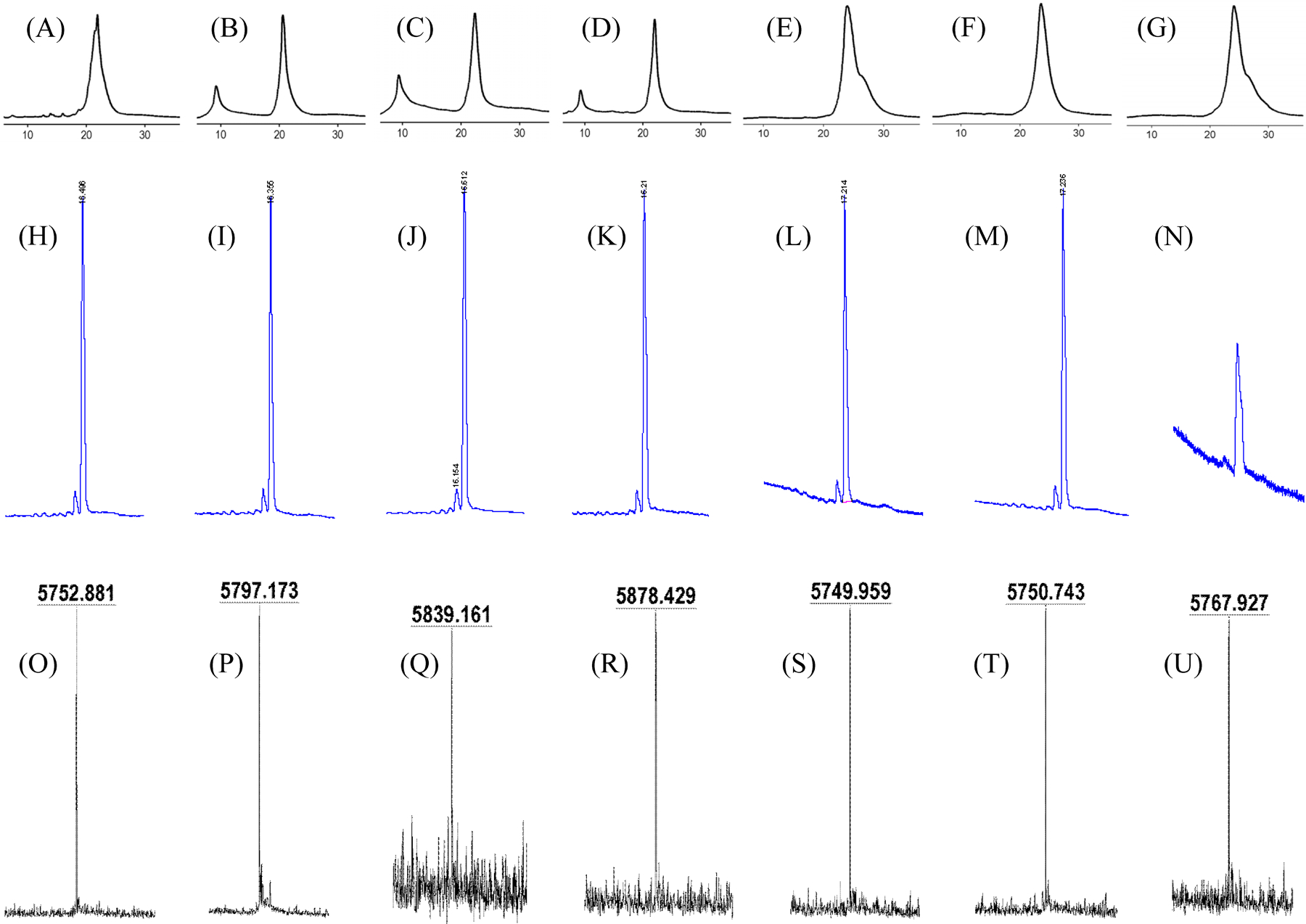
RP HPLC, capillary electrophoresis (CE), and MALDI MS of ODNs **1a**–**g**. (A-G) RP HPLC of ODNs **1a**–**g**, respectively. The peak at ~22 min is from the ODN. In some of the profiles, there is a peak at 10 min or a shoulder at the ODN peak. They are from non-ODN materials likely from the HPLC system as the CE and MALDI MS as well as gel electrophoresis (see supporting information) analyses all indicate that the ODNs are pure. (H-N) CE profiles of ODNs **1a**–**g**, respectively. (O) MS of ODNs **1a**, calcd [M−H]^−^ 5753.0, found 5752.9. (P) MS of ODNs **1b**, calcd [M+H]^+^ 5796.0, found 5797.2. (Q) MS of ODNs **1c**, calcd [M+H]^+^ 5839.0, found 5839.2. (R) MS of ODNs **1d**, calcd [M−H]^−^ 5879.0, found 5878.4. (S) MS of ODNs **1e**, calcd [M−H]^−^ 5753.0, found 5750.0. (T) MS of ODNs **1f**, calcd [M−H]^−^ 5753.0, found 5750.7. (O) MS of ODNs **1g**, calcd [M−H]^−^ 5769.0, found 5767.9.

The ODNs were purified by precipitation with *n*BuOH from water to remove small organic molecules resulted from deprotection. The precipitate was then further purified with RP HPLC, which was made possible with the Tr group at the 5′-end of the ODNs. The typical DMTr group was found unstable in the NaIO_4_ oxidation step during ODN deprotection and cleavage. The purified Tr-on ODN was then detritylated with 80% AcOH, and purified again with RP HPLC. The purity of the ODNs was evaluated by HPLC and capillary electrophoresis (CE, Figure 3) as well as polyacrylamide gel electrophoresis (supporting information). The identity of the ODNs was confirmed with MALDI MS (Figure 3). All the ODNs (**1a**–**h**) including those with modifications, once purified with RP HPLC and stored at −20 °C, were found stable for at least one day according to MALDI MS analysis, and are predicted to be stable for a much longer time.

Using the Dmoc method, ODNs **1a**–**g** (Table 1) were synthesized. The 19-mers were chosen because some oligonucleotide therapeutics are around this length, and ODNs around this length reliably give sharp peaks in MALDI MS spectra, which is important for determining if the introduced modifications are retained or lost. Among the ODNs, **1a**–**d** contain one to four 4acC modifications, respectively. As shown in Figures 3A–D, the acetylated ODNs showed a major peak in RP HPLC profiles. Unfortunately, besides the major ODN peak, the peak for **1a** had a shoulder before the major peak, and in the profiles of **1b**–**d**, a smaller peak appeared at ~10 minutes. However, we believe that these were caused by non-ODN materials from the HPLC system because CE analysis of the samples gave single sharp peaks (Figures 3H–K). In addition, the samples all gave single sharp peaks in MALDI MS with predicted molecular mass (Figures 3O–R), and gel electrophoresis analysis also gave single bands (supporting information). The MS data indicates that the 4acC modification was stable under the Dmoc ODN synthesis, deprotection and cleavage conditions. It is well known that 4acC and ac4C are highly sensitive modifications. It is remarkable that the ODN **1d**, which contained four 4acC modifications densely packed in a short sequence, could be synthesized. We also made efforts to synthesize an ODN containing five 4acC modifications. However, MALDI MS indicated that the sequence was not stable as the product was found to be contaminated with a small quantity of the sequence containing only four 4acC modifications.

The ODNs **1e**–**f** contain a 2acG and 6acA modification, respectively. As shown in Figures 3E–F, a major peak corresponding to the ODNs was observed although the peak of **1e** had a shoulder after the major peak. Again, we believe that it was caused by our HPLC system because both samples gave a single sharp peak in CE profiles (Figures 3L–M), and MALDI MS gave a single sharp peak with predicted molecular mass (Figure 3S–T). In addition, gel electrophoresis analysis gave single bands (supporting information). The 2acG and 6acA modifications have not been found in nature. However, it is possible that they behave similarly as 4acC and ac4C, and may increase ON cellular stability, duplex stability, and mRNA translational efficiency. The success of their incorporation into ODN is expected to facilitate the study of these and other biophysical properties of such ODNs. It is noted that among the ODNs containing the 4acC, 2acG, and 6acA modifications, the ODN containing 2acG is most labile. The success in synthesizing ODN **1e**, and purifying and analyzing it indicates that 2acG, like 4acC and 6acA, is stable enough for applications such as antisense, RNAi, CRISPR, and mRNA therapeutic development.

Besides incorporation of acetylated nucleosides into ODNs, the ODN **1g**, which contains the 4mcC modification was also synthesized. As shown by its HPLC (Figure 3G) and CE (Figure 3N) profiles, as well as MALDI MS (Figure 3U) and gel electrophoresis analysis (supporting information), this modification is also stable under the Dmoc DNA synthesis, deprotection and cleavage conditions. This finding is predictable because the electron density of the carbonyl carbon of 4mcC should be higher than that of 4acC. Like 2acG and 6acA, 4mcC has not been found in nature, and probably does not exist in nature. However, ODNs containing it may be useful for therapeutic development and other applications.

**Figure 4.**
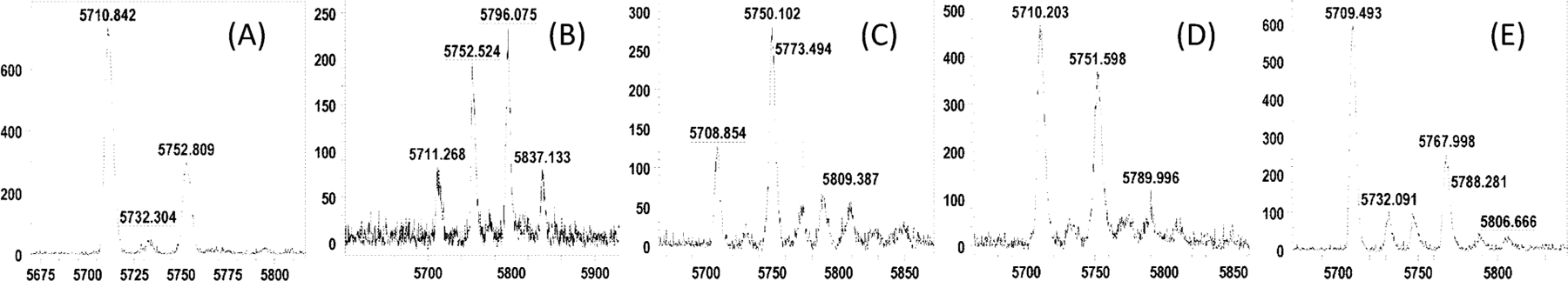
MALDI MS of ODNs **1a**–**b**, and **1e**–**g** with ODN **1h** as internal standard to indicate that the acyl groups in 4acC and 4mcC were stable under the ODN synthesis, deprotection and cleavage conditions. (A) ODN **1a**, calcd [M−H]^−^ 5753.0, found 5752.8. ODN **1h**, calcd [M−H]^−^ 5711.0, found 5710.8. The found mass difference 42.0 matches 42.0 of the acetyl group (C_2_H_2_O). The peak at 5732.3 is from the sodium adduct of **1h**. (B) ODN **1b**, calcd [M+H]^+^ 5796.0, found 5796.1. ODN **1h**, calcd [M+H]^+^ 5712.0, found 5711.3. The found mass difference 84.8 matches 84.0 of two acetyl groups. The peak at 5752.5 is from the potassium adduct of **1h** rather than **1b** losing an acetyl group because **1h** also gave the potassium adduct peak 5837.1, and in the MS of pure **1b** no corresponding peak was observed. (C) ODN **1e**, calcd [M−H]^−^ 5753.0, found 5750.1. ODN **1h**, calcd [M−H]^−^ 5711.0, found 5709.0. The found mass difference 41.1 matches 42.0 of the acetyl group. (D) ODN **1f**, calcd [M−H]^−^ 5753.0, found 5751.6. ODN **1h**, calcd [M−H]^−^ 5711.0, found 5710.2. The found mass difference 41.4 matches 42.0 of the acetyl group. (E) ODN **1g**, calcd [M−H]^−^ 5769.0, found 5768.0. ODN **1h**, calcd [M−H]^−^ 5711.0, found 5709.5. The found mass difference 58.5 matches 58.0 of the methoxycarbonyl group (C_2_H_2_O_2_). The additional minor peaks are from sodium and potassium adducts.

To further confirm that the acetyl and methoxycarbonyl groups in ODNs **1a**–**g** did not fall off from the ODNs during ODN synthesis, deprotection, cleavage, purification and analysis, the ODNs **1a**–**b** and **1e**–**g** were further analyzed with MALDI MS using the ODN **1h**, which has the same sequence as **1a**–**g** but does not have any modifications, as an internal standard. As shown in Figure 4, the mass differences of ODNs **1a**–**b** and **1e**–**g**, which contain one or more modifications, from the internal standard **1h**, which are 42.0, 84.8, 41.1, 41.4, and 58.5, respectively, matches the predicted values 42.0, 84.0, 42.0, 42.0, and 58.0. This unambiguously confirms that the Dmoc ODN synthesis method is suitable for the synthesis of sensitive ODNs containing the modifications 4acC, 2acG, 6acA, and 4mcC.

## 4. Conclusions

In summary, using the Dmoc ODN synthesis method, we were able to synthesize sensitive ODNs containing the 6acA, 2acG, and 4mcC, as well as multiple 4acC modifications without any sequence limitations. The 4acC modification is highly sensitive, and therefore the ability of the Dmoc method to incorporate four of it into one ODN sequence is remarkable. The 6acA, 2acG and 4mcC modifications have not been found in nature. However, they may behave similarly as 4acC and ac4C in terms of benefits to DNA and RNA duplex stability, and may found applications such as antisense, RNAi, CRISPR, and mRNA therapeutics. We expect that the demonstration of their incorporation into ODNs in the present work will facilitate projects aimed at studying their biophysical properties and potentials for therapeutic applications.

## 5. Patent

Sensitive Oligonucleotide Synthesis Using Sulfur-Based Functions as Protecting Groups and Linkers; U.S. Application # 16/946,455; Application Date June 23, 2020; Issuing Date December 6, 2022; Patent Number 11,518,780.

## Supplementary Material

Supplementary Information

## Figures and Tables

**Table 1.** ODN sequences, OD_260_ and mass data.

Entry	ODN	Sequences	OD_260_^[a]^	MALDI MS	

				Calculated	Found
1	**1a**	5′-TAGTA4acCTTTATCCAACCTT-3′	1.13	[M−H]^−^ 5753.0	5752.9
2	**1b**	5′-TAGTACTTTAT4acCCAA4acCCTT-3′	1.04	[M+H]^+^ 5796.0	5797.2
3	**1c**	5′-TAGTA4acCTTTAT4acCCAA4acCCTT-3′	0.53	[M+H]^+^ 5839.0	5839.2
4	**1d**	5′-TAGTA4acCTTTAT4acCCAA4acC4acCTT-3′	0.37	[M−H]^−^ 5879.0	5878.4
5	**1e**	5′-TA2acGTACTTTATCCAACCTT-3′	3.22	[M−H]^−^ 5753.0	5750.0
6	**1f**	5′-TAGT6acACTTTATCCAACCTT-3′	1.07	[M−H]^−^ 5753.0	5750.7
7	**1g**	5′-TAGTA4mcCTTTATCCAACCTT-3′	1.84	[M−H]^−^ 5769.0	5767.9
8	**1h**	5′-TAGTACTTTATCCAACCTT-3′	-	[M−H]^−^ 5711.0	-

[a] Values were based on 0.52 μmol ODN synthesis.

## Data Availability

The original contributions presented in this study are included in the article/supplementary material. Further inquiries can be directed to the corresponding author.

## Abbreviations

2acG: *N*^2^-acetylguanosine
4acC: *N*^4^-acetyldeoxycytidine
4mcC: *N*^4^-methyoxycarbonyldeoxycytidine
6acA: *N*^6^-acetyladenosine
ACN: Acetonitrile
CE: Capillary electrophoresis
CPG: Controlled pore glass
DBU: 1,8-Diazabicyclo(5.4.0)undec-7-ene
DCA: Dichloroacetic acid
DCI: 4,5-Dicyanoimidazole
DCM: Dichloromethane
Dmoc: 1,3-dithian-2-yl-methoxycarbonyl
DMTr: Dimethoxytrityl
meDmoc: Methyl Dmoc
ODN: Oligodeoxynucleotide
ON: Oligonucleotide
ORN: Oligoribonucleotide
Tr: Trityl
