# Supplementary material for: Synthesis of sensitive oligodeoxynucleotides containing acylated cytosine, adenine, and guanine nucleobases
Source: DNA (Basel). Author manuscript; Available in PMC 2025 Jul 24. (PMC12288508; doi:10.3390/dna5020025)
Supplement: Supplementary Information [file NIHMS2074416-supplement-Supplementary_Information.docx]

Supporting information

**Synthesis of sensitive oligodeoxynucleotides containing acylated cytosine, adenine, and guanine nucleobases**

Komal Chillar, Rohith Awasthy, Marina Tanasova, and Shiyue Fang*

Department of Chemistry, and Health Research Institute, Michigan Technological University, 1400 Townsend Drive, Houghton, MI 49931, USA

Email: shifang@mtu.edu

**General:** All reactions were performed in oven-dried glassware under nitrogen using standard Schlenk techniques. Reagents and solvents from commercial sources were used as received with the following exceptions. DCM and pyridine were distilled over CaH_2_ under nitrogen. THF was distilled over CaH_2_ and then Na/benzophenone under nitrogen. Sigma-Aldrich TLC plates, silica gel 60F-254 over glass support, 0.25 µm thickness, were used for TLC. Selecto Scientific silica gel, particle size 32-63 µm, was used for flash column chromatography. ^1^H, ^13^C and ^31^P NMR spectra were measured on Bruker’s spectrometer at 500, 126 and 202 MHz, respectively. Chemical shifts (*δ*) were reported in reference to solvent peaks, residue CHCl_3_ at 7.24 ppm for ^1^H, CDCl_3_ at 77.00 ppm for ^13^C, and to H_3_PO_4_ at 0.00 ppm for ^31^P. ODN synthesis was performed on a MerMade 6 solid phase synthesizer. RP HPLC was performed on a JASCO LC-2000Plus System: pump, PU-2089Plus Quaternary Gradient; detector, UV-2075Plus. Column: C-18 reversed phase, analytical, 5 μm diameter, 100 Å, 250 × 4.60 mm. Solvent A: 0.1 M triethylammonium acetate, 5% ACN. Solvent B: 90% ACN. All profiles were generated by detecting absorbance at 260 nm using the linear gradient solvent system: solvent B (0%-45%) in solvent A over 60 min followed by solvent B (45%-100%) in solvent A over 20 min at a flow rate of 1.0 mL/min. Capillary electrophoresis (CE) was carried out on an Agilent 7100 CE system with UV-Visible diode-array detector (190–600 nm) as described previously.^1^ Capillary: PVA coated 25 cm × 100 µm (the commercial 40 cm capillary was cut to 25 cm). Buffer solution: 200 mM Bis-Tris and 200 mM boric acid in CE water, pH 7.2. Sieving solution: 27% or 30% (w/v) PEG 35,000 in buffer solution. Flush regimen: High pressure flush from outlet -8 bar for 5 min. Injection: 0.02-0.07 µM 19-mer ODNs in buffer solution, -10 KV for 10 sec. Run: -25 KV, 30 ^o^C for 40 min. Detection: alignment interface for standard capillary with 75 µm ID, detected at 260 ± 8 nm. HRMS was obtained on a Thermo HR-Orbitrap Elite Mass Spectrometer. MALDI-TOF MS were obtained on Bruker’s microflex™ LRF MALDI-TOF System.

*Compound* ***7****:* 2′-Deoxycytidine (5.00 g, 22.00 mmol, 1.0 equiv.) was co-evaporated with dry pyridine four times. The compound was dissolved in the solvent mixture of dry pyridine (15 mL) and DCM (70 mL), and cooled to 0 °C. Trimethylsilyl chloride (TMSCl, 25.13 mL, 198.04 mmol, 9.0 equiv.) was added dropwise over 5 min. The reaction was allowed to proceed at rt for 2 h. After cooling to 0 °C, methyl chloroformate (1.87 g, 24.20 mmol, 1.1 equiv.) was added dropwise. The mixture was stirred for 3 h while warming to rt gradually. The mixture was cooled to 0 °C, and methanol was added to quench the reaction. Volatiles were removed under reduced pressure. The crude product **7** was used directly for the synthesis of **8**. Product **7**: pale brown oil; TLC *R*_f_ = 0.35 (SiO_2_, DCM/MeOH 5:1). The compound is known.^2^

*Compound* ***8***: DMTrCl (9.15 g, 27.00 mmol, 1.1 equiv.) was added to the solution of **7** (7.00 g, 24.55 mmol, 1.0 equiv.) in pyridine (50 mL) at 0 °C under positive nitrogen pressure. The mixture was stirred for 8 h while warming to rt gradually. The volume of the mixture was reduced to ~2 mL under vacuum generated by an oil pump (the remaining pyridine was for preventing unintended deprotection of DMTr). The mixture was partitioned between EtOAc (50 mL) and 5% Na_2_CO_3_ (50 mL). The aqueous layer was extracted with EtOAc (15 mL × 3). Organic extracts were combined, dried over anhydrous Na_2_SO_4_, filtered, and concentrated to dryness. Product **8** was purified with flash chromatography (SiO_2_, EtOAc/MeOH 18:1 with 5% Et_3_N): 4.09 g, 32% (yield based on compound **6**); pale yellow foam; TLC *R*_f_ = 0.45 (SiO_2_, EtOAc/MeOH 18:1 with 5% Et_3_N); ^1^H NMR (500 MHz, CDCl_3_) *δ* 2.21-2.26 (m, 1H), 2.77-2.82 (m, 1H), 3.39-3.49 (m, 2H), 3.77 (s, 3H), 3.79 (s, 6H), 4.20-4.21 (m, 1H), 4.53-4.55 (m, 1H), 6.31 (t, *J* = 5.8 Hz, 1H), 6.84-6.86 (m, 4H), 7.00-7.01 (m, 1H), 7.24 (d, *J* = 6.9 Hz, 1H), 7.30 (d, *J* = 7.4 Hz, 5H), 7.40 (d, *J* = 7.8 Hz, 2H), 8.24 (d, *J* = 7.1 Hz, 1H); ^13^C NMR (126 MHz, CDCl_3_) *δ* 42.1, 53.1, 55.3, 62.9, 70.9, 86.6, 86.9, 87.4, 94.9, 113.3, 127, 1, 128.0, 128.2, 129.1, 130.1, 135.5, 135.6, 144.3, 153.0, 155.4, 158.7, 162.5; HRMS (ESI) *m/z* calcd for C_32_H_33_N_3_O_8_Na^+^ [M + Na]^+^ 610.2160, found 610.2197. The compound is known.^3^

*Compound* ***9***: Diisopropylammonium tetrazolide (1.11 g, 6.51 mmol, 1.5 equiv.) and 2-cyanoethyl *N*,*N*,*N′*,*N′*-tetraisopropylphosphorodiamidite (2.07 mL, 6.51 mmol, 1.5 equiv.) were added to a solution of **8** (2.55 g, 4.34 mmol, 1.0 equiv.) in dry DCM (40 mL) at rt under positive nitrogen pressure. After stirring overnight, the mixture was concentrated to dryness under reduced pressure. Product **9** was purified by dissolving the sample in the solvent mixture of EtOAc/MeOH 9:1 with 5% Et_3_N, loading onto a column (SiO_2_), and eluting with the same solvent mixture: mixture of diastereomers; 2.64 g, 78%; white foam; TLC *R*_f_ = 0.40 and 0.45 (SiO_2_, EtOAc/MeOH 9:1 with 5% Et_3_N); ^1^H NMR (500 MHz, CDCl_3_): *δ* 1.07-1.18 (m, 1H), 2.26-2.31 (m, 1H), 2.40-2.45 (m, 0.5H), 2.75-2.78 (m, 1.5H), 3.27-3.29 (m, 0.5H), 3.37-3.40 (m, 1H), 3.48-3.53 (m, 1.5H), 7.73-3.77 (m, 4H), 3.79 (s, 3H), 3.81 (s, 6H), 4.20-4.22 (m, 1H), 4.57-4.61 (m, 1H), 6.27 (t, *J* = 6.0 Hz, 1H), 6.74-6.77 (m 2H), 6.84-6.86 (m, 3H),7.07-7.19 (m, 4H), 7.28-7.39 (m, 4H), 7.40 (d, *J* = 7.6 Hz, 1.5H), 8.21 (t, *J* = 7.4 Hz, 0.5H); ^13^C NMR (126 MHz, CDCl_3_): *δ* 20.4, 22.9, 24.6, 43.2, 45.3, 55.2, 57.8, 58.2, 60.4, 62.4, 72.6, 85.6, 86.9, 94.5, 113.3, 117.5, 127.1, 127.8, 127.9, 128.2, 128.5, 130.1, 135.2, 144.1, 152.7, 154.9, 155.3, 158.7, 162.1. The compound is known.^3^

*Compound* ***11***: Synthesized using the procedure for **7**. 2′-Deoxyadenosine monohydrates (5.00 g, 18.6 mmol, 2.0 equiv.), pyridine (15 mL), DCM (70 mL), TMSCl (21.24 mL, 167.4 mmol, 9.0 equiv.), acetic anhydride (1.94 mL, 20.46 mmol, 9.0 equiv.), and methanol (11.30 mL, 279.0 mmol, 9.0 equiv.) were used. Product **11**: brown oil; TLC *R*_f_ = 0.25 (SiO_2_, DCM/MeOH 5:1). The compound is known.^4^

*Compound* ***12***: Synthesized using the procedure for **8**. Compound **11** (2.00 g, 6.82 mmol, 1.0 equiv.), pyridine (30 mL), and DMTrCl (2.54 g, 7.50 mmol, 1.1 equiv.) were used. Product **12** was purified with flash column chromatography (SiO_2_, EtOAc/MeOH 9:1 with 5% Et_3_N): 2.92 g, 72%; colorless oil; TLC *R*_f_ = 0.5 (SiO_2_, EtOAc/MeOH 9:1 with 5% Et_3_N); ^1^H NMR (500 MHz, CDCl_3_): *δ* 2.37 (s, 3H), 2.78-2.90 (m, 1H), 3.25-3.31 (m, 0.5H), 3.36-3.44 (m, 2.5H), 3.74 (s, 6H), 4.25-4.28 (m, 1H), 4.72-4.76 (m, 1H), 6.48-6.53 (m, 1H), 6.79 (d, *J* = 7.5 Hz, 4H), 7.18-7.32 (m, 8.5H), 7.41-7.43 (m, 2H), 8.05-8.06 (m, 0.5H), 8.20-8.21 (m, 0.5H), 8.30 (s, 0.5H), 8.64 (0.5H); ^13^C NMR (126 MHz, CDCl_3_): *δ* 25.7, 40.4, 40.4, 55.3, 63.9, 72.1, 84.5, 84.9, 86.6, 113.2, 119.8, 122.4, 125.4, 127.0, 127.9, 128.3, 129.1, 129.6, 130.1, 135.8, 137.9, 139.0, 141.7, 144.7, 149.3, 149.5, 151.0, 152.3, 152.9, 155.7, 158.6, 166.1; HRMS (ESI): *m/z* calcd for C_33_H_33_N_5_O_6_Na^+^ [M + Na]^+^ 618.2323, found 618.2353. The compound is known.^5^

*Compound* ***13***: Synthesized using the procedure for **9**. Compound **12** (4.20 g, 7.05 mmol, 1.0 equiv.), DCM (50 mL), diisopropylammonium tetrazolide (1.81 g, 10.58 mmol, 1.5 equiv.), and 2-cyanoethyl *N*,*N*,*N*′,*N*′-tetraisopropylphosphorodiamidite (3.36 mL, 10.58 mmol, 1.5 equiv.) were used. Product **13** was purified by dissolving the sample in the solvent mixture of hexanes/EtOAc 1:1 with 5% Et_3_N, loading onto a column (SiO_2_), and eluting with the same solvent mixture: mixture of diastereomers; 4.15 g, 74%; pale yellow oil; TLC *R*_f_ = 0.2 and 0.3 (SiO_2_, hexanes/EtOAc 1:1 with 5% Et_3_N); ^1^H NMR (500 MHz, CDCl_3_): *δ* 1.24-1.29 (m, 12H), 1.98 (s, 3H), 2.54-2.69 (m, 2H), 2.74-2.82 (m, 3.3H), 2.95-3.03 (m, 1.7H), 3.25-3.32 (m, 2.2H), 3.40-3.51 (m, 6H), 3.65-3.75 (m, 1.8H), 3.79 (s, 6H), 4.03-4.17 (m, 3H), 4.42-4.45 (m, 1H), 5.16-5.21 (m, 1H), 6.46 (t, *J* = 6.1 Hz, 1H), 6.78-6.81 (m, 4H), 7.25-7.28 (m, 7H), 7.38 (d, *J* = 7.6 Hz, 2H), 8.00-8.02 (m, 1H), 8.27 (d, *J* = 6.0 Hz, 1H), 11.59 (b, 1H); ^13^C NMR (126 MHz, CDCl_3_): *δ* 22.5, 23.3, 46.0, 46.4, 55.2, 59.9, 60.2, 60.3, 63.4, 84.3, 85.3, 86.6, 113.1, 116.6, 116.8, 119.8, 126.9, 127.9, 128.1, 130.0, 135.4, 138.8, 144.3, 149.6, 152.7, 155.3, 158.6; ^31^P NMR (202 MHz, CDCl_3_): *δ* 148.91; HRMS (ESI): *m/z* calcd for C_42_H_50_N_7_O_7_PNa^+^ [M + Na]^+^ 818.3402, found 818. 3424. The compound is known.^6^

*Compound* ***15***: Synthesized using the procedure for **11**. The compound is known.^7^

*Compound* ***16***: Synthesized using the procedure for **8**. Compound **15** (4.00 g, 12.93 mmol, 1.0 equiv.), pyridine (50 mL), and DMTrCl (4.82 g, 14.22 mmol, 1.1 equiv.) were used. Product **16** was purified with flash column chromatography (SiO_2_, EtOAc/MeOH 8:2 with 5% Et_3_N): 4.11 g, 52%; pale yellow foam; TLC *R*_f_ = 0.20 (SiO_2_, EtOAc/MeOH 8:2 with 5% Et_3_N); ^1^H NMR (500 MHz, CDCl_3_): *δ* 2.26 (s, 3H), 2.44-2.61 (m, 2H), 3.31 (s, 2H), 3.69 (s, 6H), 4.20-4.23 (m, 1H), 4.71-4.74 (m, 1H), 6.17 (t, *J* = 6.2 Hz, 1H), 6.71 (d, *J* = 8.6 Hz, 1H), 7.11 (d, *J* = 7.2 Hz, 1H), 7.16 (t, *J* = 7.5 Hz, 2H), 7.23 (d, *J* = 8.6 Hz, 4H), 7.35 (d, *J* = 7.5 Hz, 2H), 7.87 (s, 1H); ^13^C NMR (126 MHz, CDCl_3_): *δ* 24.3, 40.7, 55.2, 64.3, 71.8, 84.5, 86.5, 86.7, 113.1, 120.8, 126.9, 127.8, 128.2, 130.1, 135.7, 138.0, 144.6, 147.9, 148.6, 156.2, 158.5, 173.7; HRMS (ESI): *m/z* calcd for C_33_H_33_N_5_O_7_Na^+^ [M + Na]^+^ 634.2272, found 634.2292. The compound is known.^8^

*Compound* ***17***: Synthesized using the procedure for **9**. Compound **16** (3.60 g, 5.89 mmol, 1.0 equiv.), DCM (50 mL), diisopropylammonium tetrazolide (1.51 g, 8.83 mmol, 1.5 equiv.), and 2-cyanoethyl *N*,*N*,*N*′,*N*′-tetraisopropylphosphorodiamidite (2.81 mL, 8.83 mmol, 1.5 equiv.) were used. Product **17** was purified by dissolving the sample in the solvent mixture of EtOAc/MeOH 9:1 with 5% Et_3_N, loading onto a column (SiO_2_), and eluting with the same solvent mixture: mixture of diastereomers; 3.20 g, 67%; off-white foam; TLC *R*_f_ = 0.20 and 0.25 (SiO_2_, EtOAc/MeOH 9:1 with 5% Et_3_N); ^1^H NMR (500 MHz, CDCl_3_): *δ* 1.07-1.14 (m, 12H), 2.1 (s, 3H), 2.30-2.55 (m, 1.5H), 2.64 (t, *J* = 6.2 Hz, 1H), 2.69-2.82 (m, 1.5H), 3.26-3.33 (m, 1H), 3.36-3.45 (m, 1H), 3.50-3.59 (m, 2.5H), 3.72 (s, 6H), 3.78-3.84 (m, 0.5H), 4.20-4.27 (m, 1H), 4.6404.78 (m, 1H), 6.21 (q, *J* = 6.5 Hz, 1H), 6.72-6.76 (m, 4H), 7.13-7.22 (m, 3H), 7.27 (t, *J* = 9.3 Hz, 4H), 7.37-7.41 (m, 2H), 7.82 (s, 0.5H), 7.84 (s, 0.5H); ^13^C NMR (126 MHz, CDCl_3_): *δ* 20.4, 23.9, 24.6, 40.0, 43.2, 55.2, 57.7, 63.6, 74.2, 84.5, 84.9, 85.8, 86.4, 113.11, 117.9, 121.5, 121.8, 126.9, 127.8, 128.0, 128.2, 130.0, 135.6, 135.8, 137.3, 137.9, 144.5, 147.5, 148.1, 155.8, 158.5, 172.6; ^31^P NMR (202 MHz, CDCl_3_): *δ* 148.03, 148.47; HRMS (ESI) *m/z* calcd for C_42_H_50_N_7_O_8_PH^+^ [M + H]^+^ 812.3531, found 812.3548. The compound is known.^9^

**References**

1. Chillar, K.; Eriyagama, A.; Yin, Y.; Shahsavari, S.; Halami, B.; Apostle, A.; Fang, S. Oligonucleotide synthesis under mild deprotection conditions. *New J. Chem.* **2023**, *47*, 8714-8722. doi:10.1039/d2nj03845e
2. Miyata, K.; Kobori, A.; Tamamushi, R.; Ohkubo, A.; Taguchi, H.; Seio, K.; Sekine, M. Conformational studies of 4-carbamoyldeoxycytidine derivatives and synthesis and hybridization properties of oligodeoxyribonucleotides incorporating these modified bases. *Eur. J. Org. Chem.* **2006**, *2006*, 3626-3637. doi:10.1002/ejoc.200501006
3. Kobori, A.; Miyata, K.; Ushioda, M.; Seio, K.; Sekine, M. A new method for the synthesis of oligodeoxyribonucleotides containing 4-n-alkoxycarbonyldeoxycytidine derivatives and their hybridization properties. *J. Org. Chem.* **2002**, *67*, 476-485. doi:10.1021/jo010813l
4. Palacio, C. M.; Sabaini, M. B.; Iribarren, A. M.; Iglesias, L. E. An efficient and mild access to acetyl protected purine nucleosides based on a chemoselective enzymatic hydrolysis. *J. Biotechnol.* **2013**, *165*, 99-101. doi:10.1016/j.jbiotec.2013.03.004
5. Alawneh, A.; Caruthers, M. Synthesis and biological activity of phosphoramidate DNA/RNA duplexes. *(2019) Patent, WO2019/241729 A1*.
6. Weinberger, M.; Berndt, F.; Mahrwald, R.; Ernsting, N. P.; Wagenknecht, H. A. Synthesis of 4-aminophthalimide and 2,4-diaminopyrimidine c-nucleosides as isosteric fluorescent DNA base substitutes. *J. Org. Chem.* **2013**, *78*, 2589-2599. doi:10.1021/jo302768f
7. Fan, Y.; Gaffney, B. L.; Jones, R. A. Transient silylation of the guanosine o6 and amino groups facilitates-acylation. *Org. Lett.* **2004**, *6*, 2555-2557. doi:10.1021/ol049096i
8. Raynaud, F.; Asseline, U.; Roig, V.; Thuong, N. T. Synthesis and characterization of o-6-modified deoxyguanosine-containing oligodeoxyribonucleotides for triple-helix formation. *Tetrahedron* **1996**, *52*, 2047-2064. doi:10.1016/0040-4020(95)01043-2
9. Miura, F.; Fujino, T.; Kogashi, K.; Shibata, Y.; Miura, M.; Isobe, H.; Ito, T. Triazole linking for preparation of a next-generation sequencing library from single-stranded DNA. *Nucleic Acids Res.* **2018**, *46*. doi:10.1093/nar/gky452


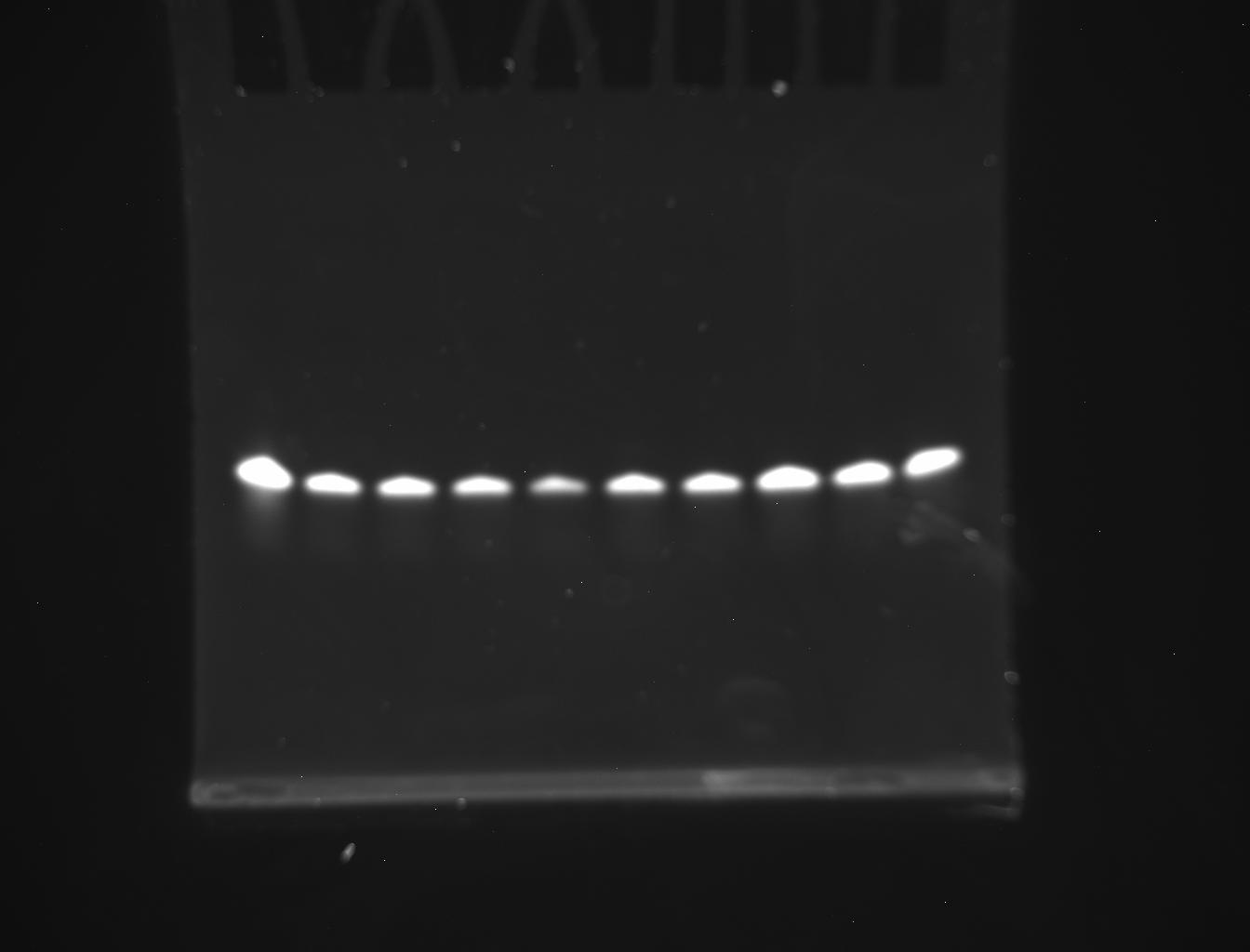


1 2 3 4 5 6 7 8 9 10

**Figure S1.** Image of denatured polyacrylamide gel electrophoresis analysis of ODNs. Polyacrylamide gel, 15%, 7 M urea, 1X TBE buffer, 35 V, 5 h. DNA 25 ng in each lane except lanes 1 and 8-10. Gel Red stain. Lane 1: Complementary strand of ODN **1h** (50 ng). Lane 2: ODN **1h**. Lane 3: ODN **1a**. Lane 4: ODN **1b**. Lane 5: ODN **1c**. Lane 6: ODN **1d**. Lane 7: a 19-mer ODN sequence with five 4acC modifications; MALDI MS indicated that this sequence was not stable due to the loss of acetyl groups. Lane 8: The mixture of ODN on lane 7 (25 ng) and ODN **1h** (25 ng). Lane 9: The ODN on lane 7 (50 ng). Lane 10: The mixture of ODN **1a** (25 ng) and ODN **1h** (25 ng). The ODNs with different modifications could not be resolved on the gel, but the single bands indicate that they do not contain any sequences that are shorter or longer than them.


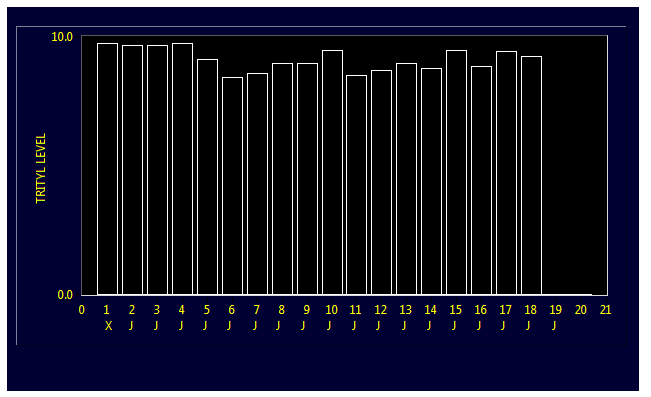


Trityl assay graph for ODN **1a** (5′-TAGTA4acCTTTATCCAACCTT-3′)


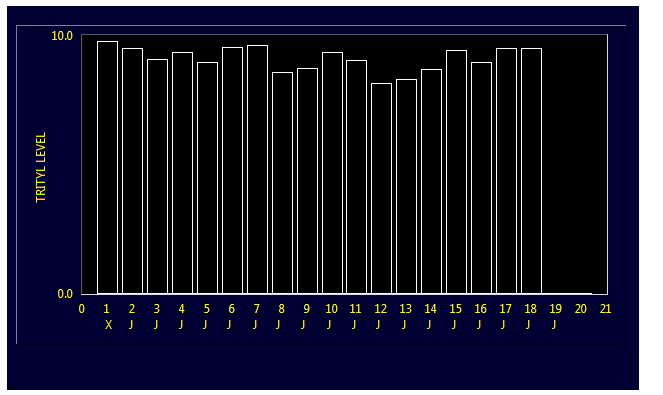


Trityl assay graph for ODN **1b** (5′-TAGTACTTTAT4acCCAA4acCCTT-3′)


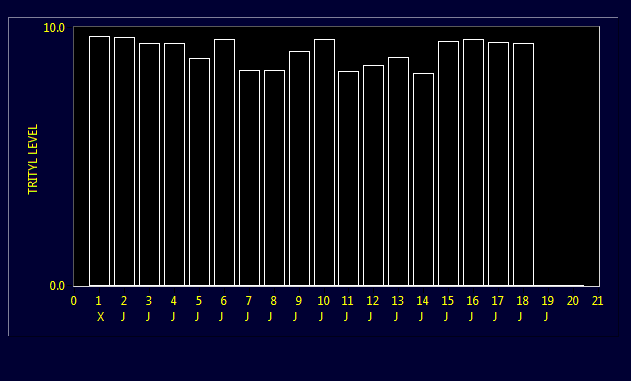


Trityl assay graph for ODN **1c** (5′-TAGTA4acCTTTAT4acCCAA4acCCTT-3′)


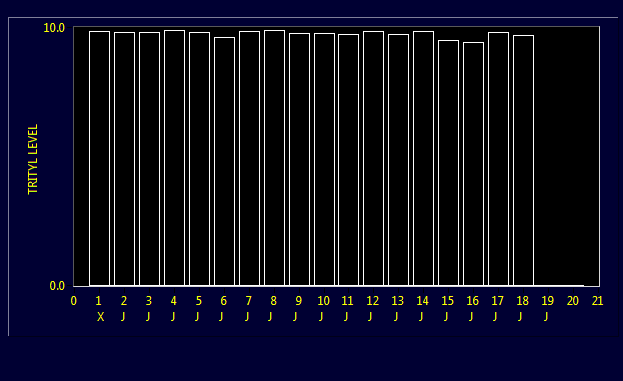


Trityl assay graph for ODN **1d** (5′-TAGTA4acCTTTAT4acCCAA4acC4acCTT-3′)


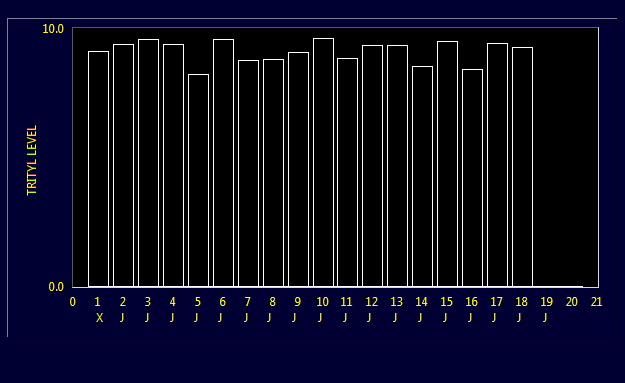


Trityl assay graph for ODN **1e** (5′-TA2acGTACTTTATCCAACCTT-3′)


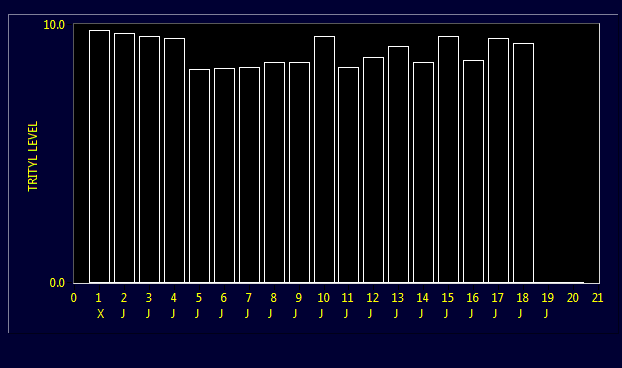


Trityl assay graph for ODN **1f** (5′-TAGT6acACTTTATCCAACCTT-3′)


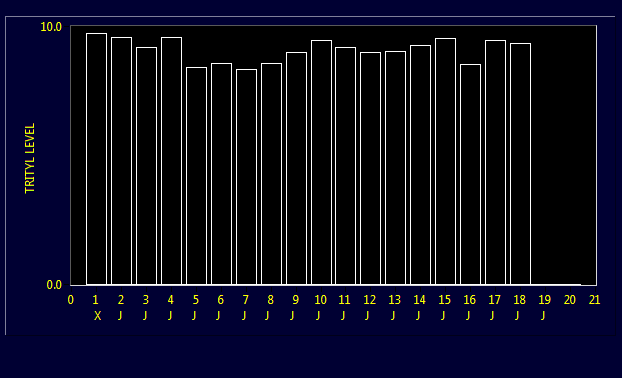


Trityl assay graph for ODN **1g** (5′-TAGTA4mcCTTTATCCAACCTT-3′)


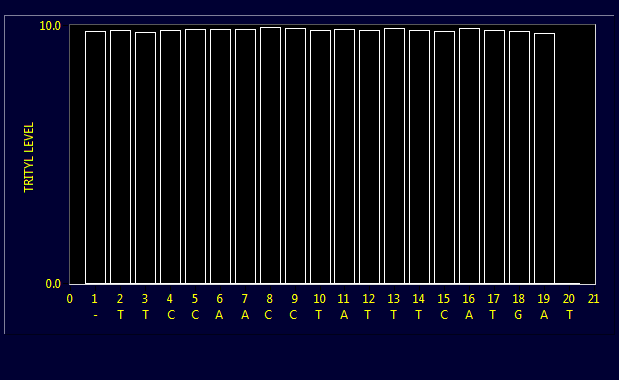


Trityl assay graph for ODN **1h** (5′-TAGTACTTTATCCAACCTT-3′)
